# Comparison of Outcomes and Costs of Ranibizumab and Aflibercept Treatment in Real-Life

**DOI:** 10.1371/journal.pone.0135050

**Published:** 2015-08-04

**Authors:** Martin K. Schmid, Oliver Reich, Livia Faes, Sophie C. Boehni, Mario Bittner, Jeremy P. Howell, Michael A. Thiel, Andri Signorell, Lucas M. Bachmann

**Affiliations:** 1 Eye Clinic, Cantonal Hospital of Lucerne, Lucerne, Switzerland; 2 Department of Health Sciences, Helsana Group, Zurich, Switzerland; 3 medignition Inc. Research Consultants, Zürich, Switzerland; Tohoku University, JAPAN

## Abstract

**Background:**

Treatment efficacy and costs of anti-VEGF drugs have not been studied in clinical routine.

**Objective:**

To compare treatment costs and clinical outcomes of the medications when adjusting for patients’ characteristics and clinical status.

**Design:**

Comparative study.

**Setting:**

The largest public ophthalmologic clinic in Switzerland.

**Patients:**

Health care claims data of patients with age-related macular degeneration, diabetic macula edema and retinal vein occlusion were matched to clinical and outcome data.

**Measurements:**

Patients’ underlying condition, gender, age, visual acuity and retinal thickness at baseline and after completing the loading phase, the total number of injections per treatment, the visual outcome and vital status was secured.

**Results:**

We included 315 patients (19595 claims) with a follow-up time of 1 to 99 months (mean 32.7, SD 25.8) covering the years 2006–2014. Mean age was 78 years (SD 9.3) and 200 (63.5%) were female. At baseline, the mean number of letters was 55.6 (SD 16.3) and the central retinal thickness was 400.1 μm (SD 110.1). Patients received a mean number of 15.1 injections (SD 13.7; range 1 to 85). Compared to AMD, adjusted cost per month were significantly higher (+2174.88 CHF, 95%CI: 1094.50–3255.27; p<0.001) for patients with DME, while cost per month for RVO were slightly but not significantly higher. (+284.71 CHF, 95% CI: -866.73–1436.15; p = 0.627).

**Conclusions:**

Patients with DME are almost twice as expensive as AMD and RVO patients. Cost excess occurs with non-ophthalmologic interventions. The currently licensed anti-VEGF medications did not differ in costs, injection frequency and clinical outcomes. Linking health care claims to clinical data is a useful tool to examine routine clinical care.

## Introduction

In Switzerland, and many countries worldwide, the two anti-VEGF medications ranibizumab and aflibercept are licensed to treat wet age-related macular degeneration and retina vein occlusion (only central vein occlusions for aflibercept), and ranibizumab is licensed for the treatment of diabetic macular edema. [1–3] Treatment efficacy and costs have been studied extensively but primarily in a stringent research setting. [4–8] From a health services research perspective, these findings cannot be translated to the clinical routine in a straightforward manner, because studied subjects tend to be selected and both the exposure and the outcomes are assessed within predefined protocols. [9]

The approach of health service research allows a different way of assessing cost and treatment efficacy, focusing on the actually applied treatment, generated financial expenses and treatment outcomes in a real-life setting. [9] With the possibility of interconnecting health care data with clinical data, a solid depiction of the actual status quo can be obtained. Provision of a status quo may reveal new information on treatment efficacy, handling and application of various medications, always put in context with the cost consequences. Subsequently, this knowledge allows adaptations to the actual routine practice, creating a base for provision of effective and sustainable medical decision-making. Therefore, we set out to compare the reimbursed treatment costs and clinical outcomes of the various regimens when adjusting for patients’ characteristics and clinical status.

## Material and Methods

This study received Ethics approval from the Ethics Commission for North-East and Central Switzerland (EKNZ 2014–110) and was adhered to Declaration of Helsinki and the principles of good clinical practice.

### Ophthalmologic management

All patients followed a pro re nata [10] treatment scheme based on optical coherence tomography (OCT) findings. Patients were followed-up on monthly basis within the retinal service of the eye clinic. At each visit, we performed a fundoscopy, measured visual acuity (ETDRS) and performed an OCT (Spectralis, Heidelberg Engineering GmbH, 69121 Heidelberg, Germany). A senior clinician made the final decision for an injection based on intra or subretinal fluid found in the OCT. The injection was made on the same day. If patients missed a follow-up visit they were approach by the clinic to agree on a new visit date. All clinical data were stored in the electronic health record system (EHRS) of the eye clinic.

### Patient identification and matching

We identified all patients with a Helsana health insurance receiving either ranibizumab or aflibercept treatments at the eye clinic using the hospital’s health claims system in the years 2006 to 2014. Of these patients we obtained all relevant clinical data from the EHRS. Helsana provided all health care claims data of these patients. The two databases were matched into one pseudonymized analysis file. To check consistency between the health claims according to the hospital’s system and the health insurer’s database, we compared data of all 19595 claims. In case of an inconsistency between dates we tried to match the clinical recordings of a specific visit to the corresponding health claim date. We accepted a discrepancy of up to 30 days between the two dates. All data management procedures were performed adhering to the current data protection protocols and the requirements of the Ethics committee. Patient information was anonymized and de-identified prior to analysis.

### Clinical parameters

Patients’ underlying condition, gender and age, the visual acuity and retinal thickness at baseline and after completing the loading phase, the total number of injections per treatment, the visual outcome and vital status was secured. There were no missing data for these parameters. If a patient provided visual acuity data on both eyes, we used the data of the eye with the longer follow-up. If the underlying condition between one of two eyes requiring anti-VEGF was different from AMD (i.e. DME or RVO), we selected that eye.

### Outcomes

The outcome parameter global costs comprised the total of health care claims for the timespan of anti-VEGF treatment in the numerator and number of months of follow-up in the denominator. The outcome parameter cost for ophthalmologic treatment comprised the total of all health care claims of the Eye Clinic that could be directly attributed to anti-VEGF therapy (i.e. drug cost, OCT, consumable material and medical consultation) in the numerator and months of follow-up in the denominator.

### Statistical analysis

We summarized continuous variates with means, standard deviations and ranges. Dichotomous variates were summarized with percentages. The association between clinical parameters and costs were first examined using univariate methods. Cost per month was computed by dividing the sum of costs (global or for ophthalmologic treatment) with the number of month in the follow-up. This cost parameters were used throughout the analyses.

We first checked whether the assumptions to use linear regression models was tenable. Normality of the error distribution was tested visually using normal probability plots and statistically using the Anderson-Darling test (null hypothesis, data is normally distributed). Based on these findings, we compared costs between different patient-groups using multivariate linear regression models where either global cost/ month or ophthalmologic cost / month were the dependent variates and two indicator variates for underlying clinical condition (AMD, DME, RVO) were the independent variates. In these models we adjusted for differences in the age distribution and frequency of female gender. The comparison between the two anti-VEGF treatments was limited to the subgroup of patients with AMD and performed using a multivariate linear regression model where ophthalmologic cost / month was the dependent and an indicator variate for de novo treatment (ranibizumab vs. aflibercept) was the independent variate. The analysis was adjusted for age, female gender, visual acuity at baseline and the number of injections. In a sensitivity analysis, we excluded the covariate for number of injections to assess, to what extent this would change the overall results. Statistical analyses were performed using the Stata 11.2 statistics software package. (StataCorp. 2009. Stata Statistical Software: Release 11. College Station, TX: StataCorp LP.)

## Results

### Patients’ characteristics

Overall, 315 patients (19595 claims) with a follow-up time of 1 to 99 months (mean 32.7; SD 25.8) qualified for inclusion. Mean age was 78 years (SD 9.3) and 200 (63.5%) were female. The majority of patients was treated due to AMD (n = 241 (81.7%)). Thirty-one patients (10.5%) had DME and 23 patients (7.8%) a RVO. In total, 270 patients (85.7%) started with and remained on ranibizumab treatment, five patients started with aflibercept *de novo* and 40 patients switched from ranibizumab to aflibercept. At baseline, the mean number of letters was 55.6 (SD 16.3) and the central retinal thickness was 400.1 μm (SD 110.1). Patients received a mean number of 15.1 injections (SD 13.7; range 1 to 85). (S1 Data)

### Comparison between clinical subgroups

Mean follow-up was longest for AMD patients (37.4 month, SD 26.6) followed by patients with DME (22.8 month, SD 15.5) and RVO (17.5 month, SD 14.5). Compared to patients with AMD (79.41 years, SD 0.53), patients with DME (- 9.92 years (SD 1.63); p<0.001) and RVO (-3.50 years (SD 1.84); p = 0.0584) were younger. Compared to patients with AMD (55.13 letters, 95% CI: 53.09 to 57.17) mean visual acuity before starting treatment was significantly higher for patients with DME (+6.53 letters 95% CI: 0.49 to 12.58; p = 0.034) and almost identical for patients with RVO (+0.17 letters, 95% CI: -6.75 to 7.08; p = 0.962). In terms of RT at baseline, the retinal layers of AMD patients were thinnest (386.71 μm, 95% CI: 373.06 to 400.35) while DME patients had +49.06 μm (95% CI: 8.64 to 89.47; p = 0.018) and RVO patients had +94.59 μm (95% CI: 48.37 to 140.82; p<0.001) thicker central retinas.

### Comparison between different treatments

The mean number of injections per month with ranibizumab and aflibercept (including the loading phase) for patients with AMD was 0.43 (SD 0.31) and 0.52 (SD 0.13), respectively (p = 0.560). Treatment intensity of patients who had switched from ranibizumab to aflibercept was significantly higher (0.68 SD 0.27; p<0.001) than among patients remaining under ranibizumab treatment. The baseline visual acuity of AMD patients receiving ranibizumab (n = 197) was 54.66 letters (SD 16.66) and 57.63 letters (SD 24.29) for patients receiving aflibercept de novo (n = 5). These differences were not statistically significant (p = 0.727). All but one patient with DME received ranibizumab throughout the follow-up.

### Assessment of costs

Mean global cost per month across all patient groups was 2023.44 CHF (SD 2684.13 CHF). The corresponding figure for ophthalmologic treatment cost per month was 1415.61 CHF (SD 957.72). Compared to AMD, total cost per month were significantly higher (+2174.88 CHF, 95%CI: 1094.50 to 3255.27; p<0.001) for patients with DME, while cost per month for RVO were slightly but not significantly higher. (+284.71 CHF, 95% CI: -866.73 to 1436.15; p = 0.627) when adjusting for patients’ age and gender. This pattern remained unchanged when only considering costs for the ophthalmologic treatment, but group differences became non-significant (AMD: 2186.98 CHF, 95% CI: 1184.58 to 3189.38); DME: + 188.13 (95% CI: -180.85 to 557.11; p = 0.316); RVO:- 0.40 CHF, 95% CI: -393.65 to 392.85; p = 0.998). Costs per month between patients receiving either ranibizumab or aflibercept (excluding patients who had switched from ranibizumab to aflibercept or vice-versa) did not differ (-264.37 CHF 95% CI: -1163.76 to 635.22; p = 0.563) when adjusting for patients’ age, female gender, baseline visual acuity and number of injections. The result, when excluding number of injections as a covariate, remained almost unchanged (-266.29 (95% CI: -1166.18 to 633.59; p = 0.560) (Table 1).

**Table 1:** Summary of cost for patient group and type of treatment.

Swiss Franc [CHF]	all (SD)	DME (vs. AMD) (95% CI)	p-value	RVO (vs. AMD) (95% CI)	p-value
**Av. global cost /month**	2023.44 (2684.13)	+2174.88 (1094.50–3255.27)	<0.001	+284.71 (-866.73–1436.15)	0.627
**Av. ophthalmologic cost /month**	1415.61 CHF (957.72	+ 188.13 (-180.85–557.11)	0.316	- 0.40 (-393.65–392.85)	0.998
		**Ranibizumab vs. Aflibercept (95% CI)**	**p-value**		
**Comparison of treatments for AMD**					
**Av. global cost /month**	1711.60 (1305.36)	-679.77* (-2052.51–692.97)	0.330		
**Av. ophthalmologic cost /month**	1350.74 (886.29)	-264.37* (-1163.76–635.22)	0.563		

*adjusted for patients’ age, female gender, baseline visual acuity and number of injections.

Over time, costs for ophthalmologic management sank by -15.83 CHF (95% CI: -19.61 to -12.05; p<0.001) per month. Monthly reductions were highest among patients with DME (-43.93 CHF, 95% CI:-66.30 to -21.57; p<0.001). The same pattern was seen for total costs (data not shown). In the subgroup of patients with AMD, the costs for ophthalmologic treatment sank by 97.23 CHF / year (95% CI -985.38 to 790.92; p = 0.829). We found no interaction between the reduction of treatment costs over time and type of treatment. Also, the results of an analysis that excluded 18 patients with short follow-up (<6 months) were not different from those of the complete group of patients.

## Discussion

### Main findings

The two currently licensed anti-VEGF medications do not differ in clinical outcomes, injection frequency and costs. Variability in costs was attributable to the underlying clinical condition, patients’ characteristics and duration of treatment. Patients with DME are almost twice as expensive as AMD and RVO patients. Cost excess in these patients compared to patients with AMD is due to non-ophthalmologic interventions. Costs for the ophthalmologic management are similar between patients with AMD, DME and RVO.

### Results in context of the existing literature

The efficacy of ranibizumab and aflibercept has been extensively studied in clinical studies. [4, 5, 7] Also, costs of treatment have been studied and cost-effectiveness analyses have been performed. [6, 8] We are unaware of any study examining clinical outcomes and costs of ophthalmologic anti-VEGF management within a health service research paradigm in a broader context. In 2013, Johnston and colleagues published the results of a retrospective analysis of first-line anti-vascular endothelial growth factor treatment patterns in AMD. [11] They used administrative claims data and compared number of injections and healthcare expenditures between patients receiving either ranibizumab or aflibercept for 6 or 12 month and found no differences between the two drugs for these two parameters. Unfortunately, they did not assess costs for patients with DME or RVO as we did. Moreover, no clinical data were available to study variability of costs within salient clinical subgroups. In respect to the equivalence of ranibizumab and aflibercept in the treatment of AMD, our findings are in an agreement. Although small differences between ranibizumab and aflibercept could be observed they are essentially equivalent both in terms of efficacy, clinical management and associated costs. Interestingly, ophthalmologic treatment costs did not differ substantially between patients with AMD, DME and RVO. This might be partly due to the fact that all three patient groups are managed within the same therapeutic concept.

### Strength and limitations

Strength includes matching economic data from health claims with clinical data. This set-up provides unique possibilities to study cost consequences of treatment in a real life setting. Moreover, access to clinical data allows studying variability of costs due to differences in the clinical profiles of patients. From a quality assurance point of view the combination of two databases allows a straightforward examination of clinical management and re-imbursement. Finally, the findings of this study allow extrapolating to the whole country and helps improving the interpretation of health claim data of the health insurer. What are the downsides of this study? Although standardized procedures for clinical management of anti-VEGF treatment have been installed at the eye clinic already in 2007, data collected in the daily routine never comply with those from clinical studies, where data collection is made according to strict protocols and care is taken to avoid missing data. Nevertheless, missing data for parameters measured were not a big problem. However, some clinical data such as findings from repeated fundus fluorescein angiography examinations, registration of reasons to switch treatment and other potentially relevant parameters were unavailable, because they are not assessed and performed in clinical routine. Therefore, our analyses could not go beyond a certain level of detail. Second, matching of the two datasets was not always straightforward. Sometimes records were ambiguous or contradictory. Although we meticulously checked for inconsistencies and cross-checked almost twenty thousand records by hand, we cannot fully rule-out that our dataset contained small errors. However, we believe that this fact does not jeopardize our overall findings. Finally, the overlap of patients treated at the eye clinic of the cantonal hospital in Lucerne and holding an insurance contract with Helsana was rather small and perhaps not perfectly representative for all Switzerland. Moreover, by only including one clinic some potential problem to generalize the findings to other settings is possible even when strictly adhering to clinical guidelines. The small number of patients with *de novo* aflibercept treatment might be an indication for this, irrespective of the short time span between licensing of aflibercept in Switzerland (2012) and the data analyzed in this study. We were unable to assess whether the strategy for each disease treatment differed during the examination periods and between the two drugs. Finally, we recognize that the duration of follow-up and the number of patients within each subgroup was sometimes limited, which had an impact on the precision of estimates but also on the generalizability of the results.

### Implications for research

We believe that research endeavors such as ours are urgently needed to improve our understanding of clinical practice. [9] We consider such work to be equally important as the research required to obtain an approval. Results from health service research studies allow validating the assumptions that were made at the time point of approval based on the results of clinical studies that may not be fully applicable to the patient domain receiving the drug after the approval. [12] Second, the collaboration between the healthcare provider, the health insurer and healthcare industry increases the understanding of all parties regarding challenges of a healthcare system. The results of studies like ours help increasing the transparency within the healthcare system which ultimately serves to the benefit of the patients.

### Implications for practice

What conclusions can be drawn from this study by the ophthalmologic clinician? At this point, the clinically most interesting finding can be attributed to the clinical and financial equality of the two treatment substances in AMD patients, supporting the findings of Johnston et al. [11] while partly challenging the findings of the VIEW-studies. [3, 13]

### Conclusions

Underlying clinical condition is a main driver for overall treatment costs. Patients with DME are almost twice as expensive as AMD and RVO patients. Cost excess occurs with non-ophthalmologic interventions. The currently licensed anti-VEGF medications did not differ in costs, injection frequency and clinical outcomes. Linking health care claims to clinical data is a useful tool to examine routine clinical care.

## Supporting Information

S1 Data The file contains the minimum dataset of 315 patients included in the analysis. (XLSX)
